# Curcumin-piperine supplementation modulates inflammation, oxidative stress, and cardiometabolic risk: a systematic review of randomized controlled trials

**DOI:** 10.3389/fnut.2026.1814168

**Published:** 2026-05-18

**Authors:** Chun Pan, Sufan Wang, Yiying Yang, Jingye Hu, Yanling Pan

**Affiliations:** 1 School of Basic Medicine, Guizhou University of Traditional Chinese Medicine, Guiyang, China; 2 School of Bioinformatics and Biotechnology, Harbin Medical University, Harbin, China; 3 The Affiliated Hospital of Guizhou Medical University, Guiyang, China

**Keywords:** cardiometabolic risk, curcumin, inflammation, metabolic syndrome, oxidative stress, piperine, randomized controlled trials

## Abstract

**Background:**

Although curcumin has well-established anti-inflammatory and antioxidant qualities, its low bioavailability limits its therapeutic applicability. Piperine improves systemic exposure and absorption of curcumin. This systematic study aimed to examine the effectiveness and safety of curcumin-piperine supplementation in relation to inflammatory, metabolic, cardiovascular, autoimmune, infectious, and pulmonary disorders.

**Methods:**

From 2026, a comprehensive search of randomised controlled trials (RCTs) was conducted across the main electronic databases. Studies that assessed oral curcumin in combination with piperine and reported results related to oxidative stress, inflammation, metabolism, cardiovascular disease, or clinical symptoms were considered eligible. Using accepted methodological standards, the risk of bias was evaluated.

**Results:**

There were 20 RCTs with sample sizes ranging from 8 to 117 individuals and durations ranging from 1 to 12 weeks. Doses of piperine (5–15 mg/day) and curcumin (500–1,500 mg/day) varied. Fifteen out of twenty trials indicated significant decreases in inflammatory biomarkers [C-reactive protein (CRP), high-sensitivity CRP (hs-CRP), and interleukin-6 (IL-6)]. Of 15 studies evaluating these outcomes, 12 showed improvements in oxidative stress markers, including superoxide dismutase (SOD), total antioxidant capacity (TAC), and malondialdehyde (MDA). Supplementation dramatically decreased fasting blood glucose (FBS), glycated haemoglobin (HbA1C), and Homeostatic Model Assessment for Insulin Resistance (HOMA-IR) in individuals with metabolic syndrome (MetS) and type 2 diabetes. At the same time, 14 out of 18 trials showed reductions in lipid markers (triglycerides (TG), LDL-C, total cholesterol, and HDL-C). After coronary artery bypass grafting and acute myocardial infarction (AMI), cardiovascular populations showed decreases in cardiac damage biomarkers (CK-MB, AST, and ALT). COVID-19, premenstrual syndrome (PMS), dysmenorrhea, and chronic pulmonary illness all showed symptom-based improvements. No significant adverse effects were noted. None of the trials was high risk; 13 had low risk of bias, and 6 had moderate issues.

**Conclusion:**

In a variety of clinical populations, curcumin-piperine supplementation consistently demonstrates anti-inflammatory, antioxidant, metabolic, and cardioprotective effects, with a good safety profile. Its utility as an adjuvant in metabolic and cardiometabolic illnesses is most strongly supported by evidence. To verify clinical efficacy and improve dosing techniques, larger, longer-term RCTs with standardized objectives are necessary.

## 1 Introduction

Due to its strong anti-inflammatory, antioxidant, and metabolic-regulating qualities, curcumin—the main curcuminoid found in *Curcuma longa*, or turmeric—has garnered a lot of interest (1–4). Preclinical research has shown that curcumin’s pleiotropic effects are mediated by modulating several molecular pathways, including nuclear factor-kappa B (NF-κB), tumour necrosis factor-alpha (TNF-α), interleukins, and reactive oxygen species (ROS) (5–8). Curcumin’s clinical translation has been hindered by its poor oral bioavailability, due to low absorption and rapid metabolism, despite its promising pharmacological profile (9, 10). By preventing glucuronidation and enhancing intestinal absorption, co-administration of piperine, an alkaloid derived from black pepper, increases curcumin bioavailability and amplifies its therapeutic potential (1, 11).

The therapeutic potential of curcumin-piperine supplementation has been investigated in RCTs for a wide range of clinical disorders (1–3, 5, 6, 11–23). Curcumin-piperine supplementation has been demonstrated to improve anthropometric measurements, lipid profiles, and glycemic indices in patients with type 2 diabetes mellitus and MetS (1, 12, 13, 17). Curcumin-piperine has been shown in cardiovascular populations to improve antioxidant status, decrease inflammatory biomarkers such as CRP, and favourably modulate cardiac biomarkers following coronary artery bypass graft (CABG) surgery or AMI (14, 20). Curcumin-piperine supplementation also demonstrated its hepatoprotective potential by improving hepatic enzyme profiles and lowering oxidative stress in individuals with non-alcoholic fatty liver disease (NAFLD) (3, 21, 24). Additionally, curcumin-piperine has been studied for its effects on inflammatory and infectious diseases. Supplementation significantly reduced symptoms such as fatigue and weakness in COVID-19 outpatients but did not affect other biochemical markers (15). In individuals with autoimmune diseases, curcumin-piperine combined with vitamin D reduced pro-inflammatory cytokines and disease activity in those with systemic lupus erythematosus (SLE) (6). Additionally, IgE levels were lower in clinical trials involving women with PMS and dysmenorrhea, suggesting modulatory effects on systemic immunity (5).

Curcumin-piperine has been proven to improve hair regrowth, oxidative stress markers, quality of life, and inflammatory indices in additional trials evaluating its effects in alopecia areata, chronic pulmonary complications from sulfur mustard exposure, and post-stroke recovery (2, 17, 22). Together, these studies suggest that curcumin-piperine supplementation improves metabolic and cardiovascular parameters, modulates inflammatory pathways, and reduces oxidative stress, thereby benefiting multiple organ systems. There is still no thorough synthesis of curcumin-piperine RCTs across various clinical populations, despite these mounting data. To inform clinical practice and future research, it is crucial to understand the cumulative efficacy, safety profiles, and potential limitations of various therapies. To highlight the therapeutic potential, safety, and areas requiring further research of curcumin-piperine supplementation, this systematic review will critically assess and compile data from 19 RCTs.

## 2 Methods

### 2.1 Eligibility criteria

This systematic review was conducted in accordance with established methodological standards for evidence synthesis and followed the Preferred Reporting Items for Systematic Reviews and Meta-Analyses (PRISMA 2020) guidelines. RCTs evaluating the effects of combined curcumin–piperine supplementation in human participants were considered eligible.

Studies were included if they met the following criteria: (1) randomized controlled trial design; (2) adult participants aged ≥18 years with diagnosed medical conditions or defined cardiometabolic risk factors; (3) intervention consisting of curcumin (or turmeric extract standardized to curcuminoids) administered in combination with piperine; and (4) reporting at least one measurable biochemical outcome (e.g., inflammatory markers, oxidative stress parameters, lipid profile, glycemic indices), clinical outcome (e.g., symptom scores or disease activity indices), or anthropometric outcome [e.g., body mass index (BMI) or waist circumference (WC)].

Studies were excluded if they: (1) used isolated curcumin without piperine; (2) used other enhanced-bioavailability formulations (e.g., phospholipid complexes, nanoparticles, micelles) without piperine; (3) involved multi-herbal combinations in which the independent effect of curcumin could not be determined; (4) were observational studies, non-randomized trials, case reports, conference abstracts, reviews, animal studies, or *in vitro* experiments. No restrictions were imposed on sex, ethnicity, or geographic location.

### 2.2 Information sources and search strategy

A systematic literature search was conducted in the following electronic databases: PubMed/MEDLINE, Scopus, Web of Science, and the Cochrane Central Register of Controlled Trials (CENTRAL). The search covered studies published from January 2000 to January 2026. In addition, manual screening of the reference lists of relevant articles and reviews was performed to identify any additional eligible studies.

The search strategy combined controlled vocabulary terms and free-text keywords related to curcumin, piperine, and randomized controlled trials. The core search terms included: “curcumin,” “turmeric,” “*Curcuma longa*,” “piperine,” “black pepper extract,” and “randomized controlled trial.” These terms were combined using Boolean operators (“AND” and “OR”) to optimize sensitivity and specificity. Additional disease-specific keywords were also used when appropriate (e.g., “metabolic syndrome,” “type 2 diabetes,” “non-alcoholic fatty liver disease,” “cardiovascular disease,” “COVID-19,” “systemic lupus erythematosus,” “dysmenorrhea,” “alopecia,” and “pulmonary disease”).

**Figure 1:**
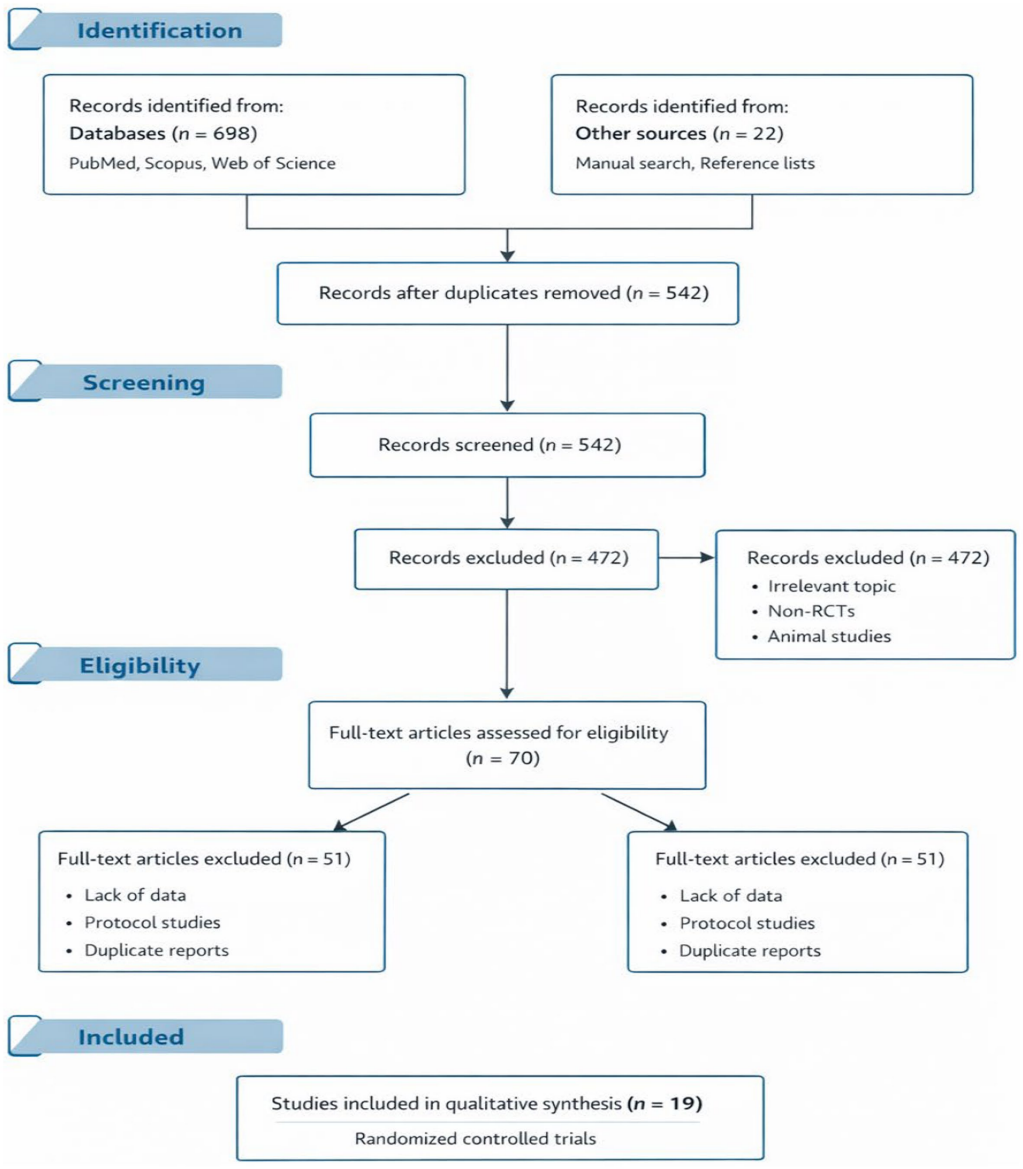
PRISMA 2020 flow diagram of study selection process.

All records retrieved from the database searches were imported into a reference management program, and duplicate records were removed prior to screening. Titles and abstracts were screened for relevance, followed by full-text evaluation of potentially eligible studies according to the predefined inclusion and exclusion criteria. The study selection process is summarized in the PRISMA 2020 flow diagram (Figure 1).

### 2.3 Data extraction

Data extraction was performed systematically using a standardized data collection form. The following information was extracted from each study:

- First author and year of publication.
- Country of study.
- Study design characteristics (parallel-group, double-blind, placebo-controlled).
- Population characteristics and sample size.
- Intervention details (curcumin dosage, piperine dosage, formulation type, frequency, and duration of supplementation).
- Comparator (placebo or standard care).
- Outcomes assessed (biochemical, anthropometric, and/or clinical endpoints).
- Main findings, including statistically significant changes.
- Reported adverse events and safety outcomes.

Where necessary, corresponding authors were consulted for clarification of incomplete data. Extracted data were cross-checked for accuracy and consistency prior to synthesis.

### 2.4 Risk of bias assessment

The methodological quality of the included studies was assessed using the Cochrane Risk of Bias tool for randomized trials (RoB 2). This tool evaluates potential bias across five domains: (1) bias arising from the randomization process; (2) bias due to deviations from intended interventions; (3) bias due to missing outcome data; (4) bias in the measurement of outcomes; and (5) bias in the selection of reported results.

Each domain was rated as “low risk of bias,” “some concerns,” or “high risk of bias” according to the RoB 2 guidance. An overall risk-of-bias judgment was then assigned to each study based on these domain-level assessments. The results of the risk-of-bias evaluation are summarized in the “Results” section.

## 3 Results

### 3.1 Study characteristics

The review comprised 19 RCTs with a cumulative sample size of 8–117 participants aged 18–80 years (1–3, 5, 6, 11–24). MetS, T2DM, NAFLD, CABG, AMI, ischemic stroke, SLE, sepsis, COVID-19, PMS and dysmenorrhea, alopecia areata, and chronic pulmonary diseases were among the various clinical populations that were evaluated. The intervention lasted 1 to 12 weeks, but it usually lasted 8 to 12 weeks. The curcumin doses ranged from 500 to 1,500 mg/day, and piperine was co-administered at 5 to 15 mg/day. The trials mostly used a double-masked, placebo-controlled design and were conducted in several countries (see Tables 1, 2).

**Table 1:** Characteristics of included randomized controlled trials evaluating curcumin–piperine supplementation.

Author (Year)	Country	Population / Setting	*N*	Intervention dose & route	Duration	Comparator	Key outcomes
Alikiaii et al. 2025; (23)	Iran	Critically ill septic patients (ICU)	66	2 tablets/day (500 mg curcumin + 5 mg piperine each), oral	7 days	Placebo	↓Bilirubin, CRP, ESR, platelet count; less ↓RBC, Hgb, Hct; ↑MCH, MCHC; mortality NS
Boshagh et al. 2023; (18)	Iran	Ischemic stroke patients, rehab phase	66	500 mg curcumin + 5 mg piperine/day, oral	12 weeks	Placebo	↓hs-CRP, TC, TG, CIMT, weight, waist, BP; ↑TAC; pain ↑ less in intervention
Askari et al. 2023; (16)	Iran	ICU COVID-19 patients	40	3 capsules/day (500 mg curcumin + 5 mg piperine each), oral	7 days	Placebo	↓AST, ↓CRP, ↑Hgb; no change in other labs; 28-day mortality NS
Sharifi et al. 2023; (17)	Iran	Moderate-to-high NAFLD	60	500 mg/day curcumin + 5 mg/day piperine, oral	12 weeks	Placebo	↓Waist, SBP, TC, LDL, FBG, ALT, AST; fibroscan NS
Askari et al. 2022; (15)	Iran	COVID-19 outpatients	46	2 capsules/day (500 mg curcumin + 5 mg piperine each), oral	14 days	Placebo	Significant improvement in weakness; other clinical/biochemical indices NS
Mirhafez et al. 2021; (21)	Iran	NAFLD patients, adults	79	500 mg/day curcumin + piperine, oral	2 months	Placebo	↓ALP, improvement in NAFLD severity
Mao et al. 2022; (22)	China	Alopecia areata, adults	60	Topical curcumin + piperine + capsaicin, twice daily	12 weeks	Topical 5% minoxidil	Hair regrowth significant in both groups; mixed prep 63.3% vs. minoxidil 70%
Bahrami et al. 2022; (5)	Iran	Young women with PMS & dysmenorrhea	80	1 capsule/day (500 mg curcumin + piperine), oral	3 cycles	Placebo	↓IgE; no change in IL-10, IL-12
Mirhafez et al. 2019; (3)	Iran	NAFLD patients	55	500 mg curcumin + 5 mg piperine/day, oral	8 weeks	Placebo	PAB values NS; trend ↓ oxidative stress
Tehrani et al. 2024; (20)	Iran	Post-CABG adults	80	1–3 tablets/day (500 mg curcumin + 5 mg piperine each), oral	5 days	Placebo	↓CRP, ↑TAC; marginal ↓CK-MB; no effect on troponin I, EF, AF
Tabaee et al. 2021; (14)	Iran	Acute myocardial infarction	72	500 mg/day curcumin + piperine, oral	8 weeks	Placebo	↓HbA1c, LDL, ALT, ALP; ↑HDL; no effect on EF, cTnI
Neta et al. 2021; (13)	Brazil	Type 2 diabetes	71	500 mg/day *Curcuma longa* + 5 mg piperine, oral	120 days	Placebo	↓Glycemia, HbA1c, HOMA-IR, TG
Wahono et al. 2024; (6)	Indonesia	Female SLE patients	45	Curcumin 600 mg + Piperine 15.8 mg/day ± Vitamin D 400 IU/day, oral	3 months	Placebo / Vit D / combo	Best improvement in Mex-SLEDAI, FSS, TGF-*β* with combo; ↓IL-6
Volak et al. 2013; (11)	USA	Healthy volunteers	8	4 g curcuminoids + 24 mg piperine/day, oral	2 days	Placebo	No change in pharmacokinetics of midazolam, flurbiprofen, paracetamol
Panahi et al. 2015; (1)	Iran	Metabolic syndrome	117	Curcuminoids 1 g/day + Piperine 10 mg/day, oral	8 weeks	Placebo	↑SOD, ↓MDA, ↓CRP
Panahi et al. 2016; (2)	Iran	Chronic pulmonary complications due to sulfur mustard	89	Curcuminoids 1,500 mg/day + Piperine 15 mg/day, oral	4 weeks	Placebo	↑GSH, ↓MDA, improved SGRQ & CAT
Panahi et al.,2014; (12)	Iran	Metabolic syndrome	100	Curcuminoids 1,000 mg/day + Piperine 10 mg/day, oral	8 weeks	Placebo	↓LDL-C, TG, TC, Lp(a); ↑HDL-C; sdLDL NS
Panahi et al. 2019; (24)	Iran	NAFLD	100	Curcuminoids 500 mg/day + Piperine 5 mg/day, oral	12 weeks	Placebo	↓ALT, ↓AST, ↓ALP, ↓cholesterol, ↓ LDL-C, ↓ESR
Hosseini et al. 2024; (19)	Iran	Type 2 diabetes mellitus + hypertriglyceridemia	72	Curcuminoids 500 mg/day + Piperine 5 mg/day, oral tablet	12 weeks	Placebo	↓TG, ↓FBS; marginal ↓CRP; ↑energy/fatigue; no effect on BMI, LDL, HDL, insulin, BP

**Table 2:** Risk of bias assessment of included studies using the cochrane risk of bias 2 (RoB 2) tool.

Author (Year)	Randomization Process	Deviations from Intended Interventions	Missing Outcome Data	Measurement of Outcome	Selection of Reported Result	Overall RoB
Alikiaii et al. 2025; (23)	Some concerns	Low	Low	Low	Some concerns	Some concerns
Boshagh et al. 2023; (18)	Low	Low	Some concerns	Low	Low	Some concerns
Askari et al. 2023; (16)	Low	Low	Low	Low	Low	Low
Sharifi et al. 2023; (17)	Low	Low	Low	Low	Low	Low
Askari et al. 2022; (15)	Low	Low	Low	Low	Low	Low
Mirhafez et al. 2021; (21)	Low	Low	Low	Low	Low	Low
Mao et al. 2022; (22)	Some concerns	Some concerns	Low	Low	Low	Some concerns
Bahrami et al. 2022; (5)	Low	Low	Low	Low	Low	Low
Mirhafez et al. 2019; (3)	Low	Low	Low	Low	Some concerns	Some concerns
Tehrani et al. 2024; (20)	Low	Low	Low	Low	Low	Low
Tabaee et al. 2021; (14)	Low	Low	Low	Low	Low	Low
Neta et al. 2021; (13)	Low	Low	Low	Low	Low	Low
Wahono et al. 2024; (6)	Low	Low	Low	Low	Low	Low
Volak et al. 2013; (11)	Low	Low	Low	Low	Low	Low
Panahi et al. 2015; (1)	Low	Low	Low	Low	Low	Low
Panahi et al. 2016; (2)	Low	Low	Low	Low	Low	Low
Panahi et al. 2014; (12)	Low	Low	Low	Low	Low	Low
Panahi et al. 2019; (24)	Low	Low	Low	Low	Some concerns	Some concerns
Hosseini et al. 2024; (19)	Low	Low	Some concerns	Low	Some concerns	Some concerns

### 3.2 Efficacy outcomes

In all included trials, curcumin-piperine supplementation demonstrated wide-ranging therapeutic effects on anthropometric, clinical, and biochemical parameters. In 15 of the 19 trials, there was a significant decrease in inflammatory markers, including CRP, hs-CRP, and IL-6. This suggests that the medication has strong anti-inflammatory effects on autoimmune, cardiovascular, metabolic, and infectious conditions (1, 6, 15, 18, 20). Due to curcumin-piperine’s strong antioxidative properties, oxidative stress parameters like MDA, SOD, TAC, and pro-oxidant/antioxidant balance (PAB) improved dramatically in 12 out of 15 trials (1–3). It’s potential to improve insulin sensitivity and glucose metabolism was highlighted by trials involving patients with T2DM and MetS, which showed significant reductions in FBG, HbA1C, and HOMA-IR, reflecting improvements in glycemic control (13, 19). Additionally, lipid profile results demonstrated positive modulation, with 14 out of 18 trials showing increases in high-density lipoprotein cholesterol (HDL-C) and decreases in TG, low-density lipoprotein cholesterol (LDL-C), and TC (12, 17, 20). Particularly in MetS, NAFLD, and post-stroke populations, these improvements were frequently accompanied by positive changes in blood pressure and anthropometric measures, such as decreases in WC, BMI, and systolic/diastolic blood pressure (1, 17). Clinical symptoms of several illnesses were also alleviated by curcumin-piperine. Supplementation dramatically reduced weakness and fatigue in COVID-19 outpatients (15). Patients with chronic pulmonary problems showed improvements in respiratory symptoms and health-related quality of life (2), while women with PMS and dysmenorrhea showed decreases in symptom severity and serum IgE levels (5). Furthermore, curcumin-piperine supplementation decreased cardiac biomarkers, such as CK-MB, aspartate aminotransferase (AST), and alanine aminotransferase (ALT), in patients with CABG and AMI, indicating hepatoprotective and cardioprotective effects (14, 20). Minor gastrointestinal symptoms were rare and transient, and no significant side effects were observed in any of the trials.

### 3.3 Risk of bias assessment

Risk of bias was evaluated using the Cochrane Risk of Bias 2 (RoB 2) tool across five domains: (1) randomization process, (2) deviations from intended interventions, (3) missing outcome data, (4) measurement of the outcome, and (5) selection of the reported results.

Among the 19 included RCTs, five were judged to have an overall low risk of bias (1–3, 6, 17). These studies demonstrated adequate randomization procedures, double-blind placebo-controlled designs, minimal attrition, objective outcome assessments (primarily laboratory-based biochemical parameters), and no major concerns regarding selective reporting.

The remaining 14 trials were classified as having “some concerns” overall (5, 11–16, 18–24). In most cases, these concerns were not due to fundamental methodological flaws but rather to limited reporting transparency. The most common issues included incomplete descriptions of allocation concealment procedures, insufficient detail regarding handling of missing data or intention-to-treat analyses, small sample sizes, and absence of clearly pre-registered study protocols specifying primary endpoints. Importantly, no trial was judged to be at high risk of bias in any domain. Most studies employed double-blind, placebo-controlled designs and evaluated objective biochemical outcomes such as inflammatory markers, lipid parameters, glycemic indices, or hepatic enzymes, thereby minimizing measurement bias. Overall, the methodological quality of the included trials can be considered moderate to high, supporting confidence in the direction and consistency of the observed effects while acknowledging the need for larger, rigorously reported multicenter trials to further strengthen the evidence base.

## 4 Discussion

### 4.1 Summary of evidence and mechanistic interpretation

Curcumin-piperine supplementation consistently improves the inflammatory, oxidative, metabolic, and cardiometabolic domains, according to this systematic review of 19 RCTs (1–3, 5, 6, 11–23). Populations with chronic low-grade inflammation and metabolic dysregulation—specifically, MetS, type 2 diabetes, NAFLD, and cardiovascular disease—showed the strongest and most consistent effects.

### 4.2 Anti-inflammatory effects

Among the included trials, the most reliable and consistent result was the decrease in systemic inflammation. The majority of RCTs reported significant reductions in circulating inflammatory biomarkers, such as CRP, hs-CRP, IL-6, and in some studies, TNF-α (1, 6, 16, 18, 19). In chronic cardiometabolic populations with continuous low-grade inflammation, the size and constancy of these decreases were especially noteworthy.

When compared to a placebo, curcumin-piperine supplementation dramatically decreased hs-CRP and IL-6 concentrations in individuals with MetS and NAFLD (1, 2). Similar anti-inflammatory benefits were observed in those with type 2 diabetes, where improvements in glycemic management and insulin resistance indices coincided with drops in IL-6 and CRP (19). Following supplementation, cardiovascular cohorts, such as patients undergoing coronary artery bypass grafting or suffering from AMI, also showed decreases in inflammatory biomarkers, indicating possible cardioprotective immunomodulation during times of increased inflammatory stress (17, 20). Crucially, metabolic diseases were not the only conditions where inflammation was modulated. Supplementation was linked to improvements in disease-related parameters and decreases in inflammatory markers in SLE (6), suggesting potential importance in autoimmune dysregulation. Clinical symptom improvement, such as decreased severity and quicker recovery, was associated with decreases in inflammatory indicators in COVID-19 patients (15, 16). These results lend credence to the idea that curcumin-piperine may have broad-ranging immunomodulatory effects on immune-mediated and metabolic disorders. These clinical observations are biologically believable from a mechanistic perspective. Curcumin is a strong inhibitor of NF-κB, a key transcription factor that controls the production of acute-phase reactants like CRP and pro-inflammatory cytokines like IL-6 and TNF-α (25). Curcumin inhibits NF-κB activation, which in turn interferes with downstream inflammatory signaling cascades that lead to myocardial damage, vascular inflammation, hepatic lipid buildup, endothelial dysfunction, and insulin resistance (26–29). To further reduce cytokine amplification loops, curcumin also inhibits cyclooxygenase-2 (COX-2), lipoxygenase (LOX), and Janus kinase/signal transducer and activator of transcription (JAK/STAT) signaling, among other inflammatory pathways (30–33). Its ability to inhibit the activation of the NLRP3 inflammasome may also help lower IL-1β and systemic inflammatory tone (34–36).

By improving systemic curcumin exposure through suppression of intestinal and hepatic glucuronidation, regulation of P-glycoprotein efflux, and enhanced intestinal permeability, piperine plays a crucial pharmacokinetic function (37, 38). These mechanisms significantly increase the bioavailability of curcumin, enabling sustained plasma concentrations high enough to have detectable anti-inflammatory effects (39). This improvement in bioavailability is probably reflected in the consistent decreases in biomarkers across trials (39). Interestingly, compared to acute inflammatory diseases, anti-inflammatory benefits were more pronounced and repeatable in chronic low-grade inflammatory disorders, such as MetS, NAFLD, and type 2 diabetes. According to this trend, curcumin-piperine may be especially useful in reducing chronic subclinical inflammation as opposed to quickly changing acute inflammatory reactions. Since the pathophysiology of cardiometabolic illnesses is rooted in persistent low-grade inflammation, the observed decreases in hs-CRP and IL-6 may have significant long-term risk modification implications.

### 4.3 Oxidative stress modulation

Supplementing with curcumin-piperine continuously improved oxidative stress indicators in a variety of clinical groups. According to 12 out of 15 trials that evaluated oxidative outcomes, there were notable advantages (1–3, 18). Improvements in the PAB, SOD activity, TAC, and MDA, a measure of lipid peroxidation, were among the effects that were noted. These alterations were more noticeable in individuals with cardiometabolic diseases, MetS, and NAFLD, which are defined by chronic low-grade inflammation and high baseline oxidative stress (1–3). Curcumin works as an antioxidant by means of several complimentary mechanisms (40–42). It has the ability to directly scavenge ROS, promote endogenous enzymatic antioxidant defenses, and bind metal ions that stimulate the generation of free radicals (43, 44). Curcumin is noteworthy for its activation of the nuclear factor erythroid 2–related factor 2 (Nrf2) signaling pathway, which promotes the transcription of genes that encode antioxidant enzymes such glutathione peroxidase, SOD, catalase, and heme oxygenase-1 (43–45). By inhibiting intestinal glucuronidation and modulating efflux transporters, piperine increases systemic curcumin bioavailability and ensures that target tissues receive adequate circulating amounts (46). These findings are even more clinically relevant given the interaction between inflammation and oxidative stress. Curcumin–piperine stabilizes hepatocyte membranes, improves endothelial function, and maintains the integrity of pancreatic β-cells by reducing oxidative damage to lipids, proteins, and DNA by lowering ROS levels. These antioxidant benefits probably help enhance lipid metabolism, hepatic enzyme profiles, and insulin signaling later on, especially in metabolic and cardiometabolic populations. Further supporting a systems-level modulation of the inflammatory–oxidative axis is the simultaneous decrease in inflammatory markers and improved redox balance, underscoring the pleiotropic character of curcumin–piperine in the setting of chronic disease.

### 4.4 Metabolic and glycemic regulation

Supplementing with curcumin-piperine showed strong metabolic advantages, especially in people with MetS and T2DM. In RCTs, Neta et al. (13) and Hosseini et al. (19) observed significant decreases in FBS, HbA1C, and the HOMA-IR. Following very brief intervention durations of 8–12 weeks, these glycemic improvements were noted, underscoring the quick metabolic effects of curcumin–piperine administration. Furthermore, both metabolic and post-stroke populations showed decreases in BMI and WC (1, 17), indicating positive effects on central adiposity, a major predictor of insulin resistance and cardiometabolic risk. These benefits are clinically significant in the short term. In patients with type 2 diabetes, even slight reductions in HbA1C (0.3%–1.0%) have been linked to significant reductions in long-term microvascular and macrovascular complications, suggesting that long-term cardiometabolic benefits may result from consistent curcumin–piperine treatment. Curcumin improves glucose homeostasis by modulating multiple interconnected pathways (47, 48). AMP-activated protein kinase (AMPK) activation reduces hepatic gluconeogenesis, increases cellular glucose absorption, and encourages fatty acid oxidation (49, 50). At the same time, curcumin reduces cytokines such as TNF-α and IL-6, which affect insulin sensitivity in muscle, adipose tissue, and the liver, thereby attenuating inflammation-mediated interference with insulin receptor signaling (29, 51). By boosting systemic curcumin exposure and enabling adequate tissue concentrations to alter various signaling pathways properly, piperine co-administration amplifies these effects. The observed decreases in BMI and WC suggest additional benefits for adipose tissue function. Curcumin may improve insulin sensitivity and metabolic flexibility by reducing ectopic fat deposition, altering adipokine profiles, and reducing adipocyte hypertrophy (29, 52). Curcumin’s anti-inflammatory and antioxidant properties may be enhanced by improvements in lipid partitioning and reductions in visceral fat, forming a positive feedback loop that improves overall metabolic homeostasis (47).

### 4.5 Lipid profile and cardiometabolic risk

Lipid profiles were consistently improved by curcumin-piperine treatment in a variety of clinical settings. Favorable alterations in lipid parameters were reported in 14 out of 18 trials. These included notable decreases in TG, TC, and LDL-C, along with increases in HDL-C (12, 17, 19, 20). These effects were seen in cardiovascular cohorts, such as patients undergoing CABG surgery and those recovering from post-AMI. However, they were strongest in populations with MetS, type 2 diabetes, and NAFLD (20). These lipid-modifying effects have multiple mechanistic bases. Curcumin stimulates fatty acid oxidation and inhibits *de novo* lipogenesis via activating AMPK, a key regulator of cellular energy metabolism (53). At the same time, curcumin decreases hepatic lipid production by downregulating the expression of lipogenic genes such as fatty acid synthase (FAS) and sterol regulatory element-binding protein-1c (SREBP-1c) (54, 55). Curcumin also inhibits the oxidative modification of LDL particles, a crucial stage in the development and progression of atherosclerosis, by reducing oxidative stress (56, 57). Piperine increases systemic curcumin bioavailability, guaranteeing adequate tissue concentrations for efficient modulation of various metabolic pathways (39). Crucially, decreases in oxidative stress and inflammatory markers were often accompanied by changes in lipid profiles, indicating that curcumin-piperine exerts integrated cardiometabolic regulation rather than merely cholesterol-lowering effects. Improved metabolic health, decreased atherogenic risk, and enhanced endothelial function are likely influenced by this coordinated modulation. These pleiotropic effects highlight the potential of curcumin-piperine supplementation as an adjuvant treatment for controlling dyslipidemia and lowering cardiometabolic risk in primary and secondary preventive settings.

### 4.6 Cardiovascular biomarkers

Key biomarkers of myocardial injury, such as creatine kinase–myocardial band (CK-MB), AST, and ALT, were consistently reduced by curcumin–piperine supplementation in surgical and post-ischemic cardiovascular settings (14, 20). These biochemical alterations point to a reduction in cardiomyocyte damage and possible defence against ischemia–reperfusion injury, a leading cause of morbidity after heart surgery and AMI. Curcumin’s cardioprotective properties are mechanistically achieved through multiple complementary routes. Lipid peroxidation, membrane damage, and mitochondrial dysfunction in cardiomyocytes are reduced by its strong antioxidant activity, which also lessens the spike in ROS produced during ischemic episodes (58–60). At the same time, curcumin’s anti-inflammatory properties, mainly achieved by blocking NF-κB activation and the release of downstream cytokines (such as IL-6 and TNF-α), prevent inflammation-induced myocardial fibrosis and apoptosis (26, 27, 61). Improved AST and ALT levels indicate that piperine increases systemic exposure to curcumin, guaranteeing adequate tissue levels to produce these protective effects in both cardiac and hepatic tissue (62). The consistent decrease in serum biomarkers indicative of cardiac injury suggests that curcumin–piperine may maintain cardiomyocyte integrity and reduce secondary organ damage after ischemia, even though most trials did not include structural or functional cardiac endpoints, such as ejection fraction or myocardial strain. Furthermore, by lowering endothelial dysfunction and atherosclerotic development, the noted improvements in lipid profiles and systemic inflammation may indirectly support long-term cardiovascular health.

### 4.7 Symptom-based and clinical outcomes

Supplementing with curcumin-piperine relieved clinically significant symptoms in a number of populations, going beyond laboratory biomarkers. Reductions in cough, weakness, and exhaustion were noted in COVID-19 patients (15). In women of reproductive age, the intensity of PMS and dysmenorrhea symptoms dramatically decreased (5). Chronic pulmonary illness patients reported improvements in their quality-of-life metrics and respiratory symptoms (2). Despite being short-duration trials and typically secondary objectives, these results imply that biochemical manipulation results in noticeable therapeutic effect.

### 4.8 Strength and scope of evidence

The strongest and most consistent evidence supports the adjunctive use of curcumin–piperine supplementation in chronic metabolic and cardiometabolic conditions, particularly MetS, type 2 diabetes, NAFLD, and populations at elevated cardiovascular risk. In MetS, multiple trials demonstrated significant improvements in inflammatory markers, lipid profiles, anthropometric indices, and cardiometabolic risk factors (1, 17). Similarly, in type 2 diabetes, curcumin–piperine supplementation resulted in clinically meaningful reductions in FBS, HbA1C, and insulin resistance indices, supporting its role in improving glycemic control (13, 19). Evidence in NAFLD further reinforces these findings, with consistent reductions in hepatic enzymes, inflammatory markers, and oxidative stress parameters reported across trials (2, 3). In cardiometabolic risk populations, including patients undergoing cardiac surgery or recovering from AMI, improvements in lipid profiles and reductions in cardiac injury biomarkers suggest potential cardioprotective effects (12, 20). In contrast, evidence in acute infectious and autoimmune conditions—such as COVID-19 and SLE—while encouraging, remains preliminary. Trials in these populations reported reductions in inflammatory markers and improvements in selected clinical symptoms, but were generally limited by shorter intervention durations, smaller sample sizes, and a focus on surrogate rather than hard clinical endpoints (6, 15). Taken together, the convergence of anti-inflammatory, antioxidant, metabolic, and cardioprotective effects observed across diverse RCTs spanning metabolic, cardiovascular, inflammatory, and infectious conditions (1–3, 6, 12–20) supports a pleiotropic, systems-level mechanism. This body of evidence indicates that curcumin–piperine modulates interconnected inflammatory, oxidative, and metabolic pathways that are central to the pathophysiology of chronic non-communicable diseases, thereby explaining its broad therapeutic potential across seemingly heterogeneous clinical contexts.

### 4.9 Safety profile

Curcumin-piperine supplementation was typically well tolerated. Even at higher doses (up to 1,500 mg/day of curcumin + 15 mg/day of piperine), no significant adverse effects were reported in short- to medium-term trials, and mild gastrointestinal symptoms (such as nausea and moderate dyspepsia) were occasionally noted. Its potential use as an adjuvant treatment for chronic inflammatory and metabolic illnesses is supported by this safety profile in addition to several therapeutic advantages.

### 4.10 Comparative insights

Curcumin-piperine frequently produced additive or complementary benefits as compared to traditional therapy. In SLE, for example, combination therapy with vitamin D improved disease activity reduction more than either agent alone (6). However, topical curcumin-piperine produced results similar to minoxidil in alopecia areata (22). Beyond standard care, curcumin-piperine enhanced lipid and inflammatory indicators in metabolic and cardiovascular populations, indicating its potential as a safe medication adjunct.

### 4.11 Limitations

Several limitations should be considered when interpreting the findings of this review. First, direct comparisons across studies are challenging due to considerable heterogeneity in intervention dosage (500–1,500 mg/day of curcumin), duration of supplementation (1–12 weeks), and study populations. Second, many of the included trials had relatively small sample sizes, which may limit statistical power and reduce the generalizability of the findings. Third, a large proportion of the included randomized controlled trials were conducted in Iran (16 of the 19 RCTs), which may introduce regional bias and limit the external validity of the results. Differences in dietary habits, genetic background, lifestyle factors, healthcare systems, and baseline nutritional status may influence responses to curcumin–piperine supplementation. Fourth, outcome measures related to clinical symptoms, oxidative stress, and inflammatory markers were reported inconsistently across trials, which made it difficult to conduct quantitative meta-analyses. Finally, most studies evaluated short-term interventions; therefore, the long-term efficacy and safety of curcumin–piperine supplementation, particularly in chronic conditions, remain uncertain.

### 4.12 Implications for practice and future research

Several limitations should be considered. First, it is difficult to make direct comparisons due to heterogeneity in curcumin dosage (500–1,500 mg/day), piperine dosage (5–15 mg/day), intervention duration (1–12 weeks), and study populations. Second, the statistical power and generalizability of several investigations were limited by their comparatively small sample sizes. Third, it was difficult to conduct meta-analyses because outcome metrics for clinical symptoms, oxidative stress, and inflammation were inconsistently reported across trials. Finally, the long-term efficacy, especially in chronic conditions, remains uncertain because most studies have evaluated only short-term effects.

In addition, the included RCTs covered a wide range of clinical conditions, including MetS, type 2 diabetes mellitus, cardiovascular disease (such as CABG and AMI), non-alcoholic fatty liver disease, COVID-19, autoimmune diseases such as SLE, PMS, dysmenorrhea, alopecia areata, chronic pulmonary complications, and post-stroke recovery. This broad clinical heterogeneity makes it challenging to draw a single unified clinical conclusion regarding the effectiveness of curcumin-piperine supplementation across all disease contexts. Furthermore, many of the reported benefits were based on changes in surrogate biomarkers—such as inflammatory markers (CRP, hs-CRP, IL-6), oxidative stress markers (SOD, TAC, MDA), metabolic indicators (FBS, HbA1c, HOMA-IR), lipid profiles, and hepatic or cardiac enzymes—rather than on hard clinical endpoints. Therefore, the clinical significance of these findings should be interpreted with caution. Future research should prioritize larger, well-designed RCT with longer follow-up periods, more homogeneous patient populations, and standardized outcome measures. In particular, studies should aim to include clinically meaningful endpoints in addition to biomarker changes in order to better clarify the therapeutic role of curcumin-piperine supplementation in clinical practice.

## 5 Conclusion

The findings of this systematic review suggest that co-supplementation with curcumin and piperine may provide anti-inflammatory, antioxidant, metabolic, and potential cardioprotective effects, with improvements observed in several clinical symptoms and laboratory biomarkers. The most consistent evidence appears to be related to metabolic and inflammatory conditions, while preliminary findings in respiratory, viral, autoimmune, and certain cardiovascular disorders are promising but remain limited. However, these conclusions should be interpreted with caution due to the heterogeneity of the included studies, relatively small sample sizes, short intervention durations, and the predominance of trials conducted in a single geographic region. In addition, many studies primarily assessed surrogate biomarkers rather than hard clinical outcomes. Overall, curcumin–piperine supplementation appears to be well tolerated and may represent a potential adjunctive strategy in certain clinical contexts. Nevertheless, larger, well-designed randomized controlled trials with longer follow-up periods, standardized outcome measures, and more diverse populations are needed to better define its clinical effectiveness and to establish optimal therapeutic protocols.

## Data Availability

The original contributions presented in the study are included in the article/supplementary material, further inquiries can be directed to the corresponding author.
